# Wild Edible Plants and Indigenous Knowledge in Urban and Rural Communities of Gondar Administrative City, Northwest Ethiopia: A Comparative Study

**DOI:** 10.1155/ijfo/4368425

**Published:** 2026-11-14

**Authors:** Tiruye Ayenew, Abiyu Enyew, Getinet Masresha, Worku Misganaw, Daniel Tadesse, Eshete Kassaw, Birtukan Asrie, Yirgalem Melkamu

**Affiliations:** ^1^ Department of Biology University of Gondar Gondar Ethiopia uog.edu.et; ^2^ Department of Biology Debark University Debark Ethiopia dku.edu.et; ^3^ Department of Plant Sciences University of Gondar Gondar Ethiopia uog.edu.et; ^4^ Department of Forestry University of Gondar Gondar Ethiopia uog.edu.et

**Keywords:** edible plants, indigenous knowledge, rural communities, urban communities

## Abstract

Wild edible plants are plant species that are used as a food source for humans without being cultivated or domesticated. They have a critical role in ensuring food security and improving nutrition in the diets of Ethiopian people. However, ethnobotanical documentations in the urban and semi‐urban areas of the country are highly limited. Thus, this study was aimed to document wild edible plants and the associated indigenous knowledge in Gondar Administrative City with particular emphasis on comparing urban and rural communities. Six urban and six rural kebeles were selected using purposive sampling method. A total of 180 respondents (120 general and 60 key informants) were selected using simple random and purposive sampling techniques, respectively. Data on ethnobotany was gathered through individual interviews, focus group discussion, guided field walk, and market assessments. Data analysis involved descriptive statistics, Jaccard’s Coefficient of Similarity (JCS), preference ranking, and triadic comparison. Sociodemographic influence on knowledge about edible plants was also assessed using SPSS Version 26.0. Thirty‐nine wild edible plants belonging to 32 genera and 23 families were identified. Trees and shrubs formed the predominant plant forms each representing 30.77%, while Fabaceae was the most prevalent family with 17.95%. The species are widely distributed in wild habitats. Significant knowledge variation on wild edible plants between rural and urban kebeles (*p* < 0.001), sex groups (*p* < 0.001), and key and general informants (*p* < 0.005) was observed. In addition, there was a positive correlation between knowledge and age of respondent (r = 0.58). *Syzygium guineense* was the most preferring and relaxing food plant that provided considerable comfort after consuming it, while *Cordia africana* was the sweetest. Fruits were the most useful edible plant parts and fresh raw form was the most widely used mode of consumption. Majority of edible plants had supplementary role and are mainly accessible in June and May. Apart from their edible value, 92.31% of plants had diversified purposes. In general, Gondar City Administration is rich with wild edible plants, which have their own contribution in improving food insecurity. These findings underscore the urgent need for integrating wild edible plants into urban biodiversity conservation strategies and food security policies in Ethiopia.

## 1. Introduction

Wild edible plants (WEPs) are plant species that are neither cultivated nor domesticated, but are available from their wild natural habitat and used as sources of food [1–4, 6]. They play a critical role in ensuring food security and improve nutrition in the diets for numerous communities around the world [7]. Even though communities across the world primarily rely on crop plants, the tradition of consuming WEPs for their nutritional role and health benefits is being reported [2, 8–10]. This is a very usual practice in developing nations such as Ethiopia where some portion of their food needs comes from non‐domesticated plants [2, 11].

In Ethiopia, WEPs are reported to be used as supplementary, seasonal, or survival food sources, and hence plays a role in combating food insecurity [2, 12, 13]. The local people have a wider tradition, knowledge and chance of using WEPs in parallel to their multi‐cultural and ethnic diversity [14]. The diverse physiogeographical features with rich plant resources also permitted the country to develop high indigenous knowledge of WEPs [15]. Previous study report [1] revealed that about 8% of the nearly 7000 higher plant species of Ethiopia are edible. In 2011, only 413 (5.9%) of them were reported to be documented [2]. However, recent systematic study [16] indicated that 951 WEPs are recorded in the country, which is accounted to 9.3% of the total estimated higher plant species of the country. But this number is still recommended as low due to incomplete study area coverage in the country [16]. In addition, the limited ethnobotanical studies, which were performed before are largely focused on rural areas, overlooking urban and semi‐urban centers; to cite some, Hassen [17], Berihun and Molla [18], Masresha et al. [19], and Awoke A et al. [20]. Comprehensive search through PubMed and Google Scholar showed that only two urban ethnobotanical studies on WEPs of Ethiopia are conducted [8, 21]. In contrary, diversity of WEPs and the associated indigenous knowledge is being lost prior to proper documentation both in urban and rural areas of the country as a result of different anthropogenic factors [22–24]. This indigenous knowledge loss had negative impact on the use of wild edible products [25]. Thus, a systematic preservation of the WEPs and the associated indigenous knowledge before its total loss is potentially valuable for future generations [1, 8, 23].

Despite its distinctive characteristic as both urban and rural center with rich historical legacy and diversified vegetation, Gondar Administrative City is one area of the nation where no ethnobotanical investigation on WEPs has carried out. Previous fieldwork and reconnaissance surveys revealed that the consumption of WEPs in the city and its rural environs is still influenced by residents’ customs and habits [26]. However, a variety of causes, including modernization and the growing demand for charcoal and firewood, are reducing the area’s overall vegetation and indigenous knowledge. Therefore, it is sensible and timely to document WEPs with the related indigenous knowledge in Gondar Administrative City before they are completely lost. Furthermore, the findings of the study can be invaluable information for relevant bodies to formulate suitable management measures. Therefore, the objective of this study was to document WEPs and the associated indigenous knowledge so as to provide insights for future conservation measures.

## 2. Materials and Methods

### 2.1. Study Area Description

Gondar Administrative City is positioned in Northwestern parts of Ethiopia at 727 km from Addis Ababa, the capital city, in between 12°29′0″N–12°40′0″N latitudes and 37°20′30″E–37°31′30″E longitudes (Figure 1). It was the former capital city of Ethiopia established in 1636 by Emperor Fasiledes and home of Fasil castle, which is a recognized UNESCO world heritages site [26, 27]. Now, it is a City Administration and also capital city of Central Gondar Zone comprised of six sub‐cities having 25 urban and 11 rural kebeles. It covers a total area of about 292.80 km^2^ with undulating mountainous topography with average elevation of 2200 meter above sea level [26–28]. Fifteen years metrological data (2010–2024) were obtained from Ethiopian Metrological Agency, Bahir Dar sub‐station, and analyzed using R software (version 4.3.3). Accordingly, the minimum and maximum temperature of the city is 11.4°C and 31.8°C, respectively, with a mean of 21.2°C and the mean annual rainfall is 1232 mm with a unimodal rainfall pattern. The population is projected to 465,973 (228,306 male and 237,667 female) primarily adhering to Ethiopian Orthodox Christianity (84.15%) [29]. Dry evergreen afromontane forest make up the majority of the natural vegetation in the city and its environs. But in recent periods, most of the natural vegetation is restricted only on Ethiopian Orthodox Tewahido church compounds.

**Figure 1:**
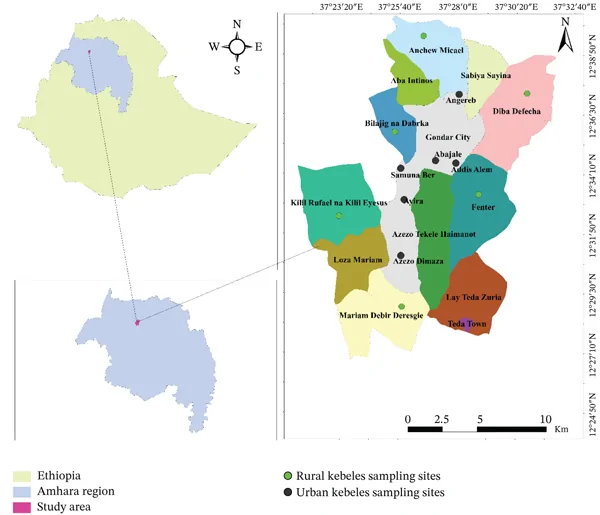
Map of the study area showing each sample site.

### 2.2. Study Site and Informant Sampling Techniques

Reconnaissance survey was done from September 12 to 19, 2025 in order to get general impression about the study area and to identify the study sites following Martin [30]. Following the survey, the sample size of 12 kebeles, equally divided between rural and urban locations, was nominated using a purposive sampling method. The selected kebeles constituted one‐third of all kebeles in the research area and took into account factors like the availability of experienced and knowledgeable practitioners, type of settlement, and where the respondents come from (Figure 1). Because of the uncertainty in determining the number of households, the sample size to be surveyed was determined through iteration, until no further beneficial information could be obtained. Consequently, the study incorporated 180 participants ranging in age from 20 to 87 years distributed equally in each sampling location (Table 1). These included 120 participants who recruited through random selection to form the general informants, while the other 60 participants selected through purposive selection to be key informants on the advice of local authorities and community elders [30, 31]. Before the interview started, each participant was aware of the study’s purpose. Following that, data collection was carried out in accordance with the International Society of Ethnobiology (ISE) Code of Ethics 2006 (http://ethnobiology.net/code-of-ethics/).

**Table 1:** Sociodemographic profile of informants.

Study site	Sample kebele	Latitude	Longitude	Altitude	Ecology	Sex		Age		Religion	
						M	F	Y	E	Ort	Isl
Urban	Angereb	12^0^37′21″	37^0^29′15″	2257	Highland	8	7	10	5	14	1
	Abajlie	12^0^36′43″	37^0^29′0″	2216	Highland	9	6	7	8	9	6
	Addis Alem	12^0^35′32″	37^0^28′50″	2104	Highland	7	8	9	6	8	7
	Qeha Eyesus	12^0^37′19″	37^0^27′19″	2179	Highland	9	6	8	7	15	—
	Samuna Ber	12^0^35′20″	37^0^26′26″	2147	Highland	6	9	11	4	14	1
	Ayira	12^0^34′58″	37^0^27′20″	2114	Highland	7	8	6	9	13	2
Rural	Diba Defecha	12^0^36′24″	37^0^30′33″	2333	Highland	7	8	5	10	13	2
	Sayina Sabiya	12^0^39′40″	37^0^30′49″	2497	Highland	8	7	6	9	12	3
	Aba Entoniyos	12^0^38′21″	37^0^27′11″	2278	Highland	9	6	6	9	15	—
	Bilajig Dabirka	12^0^36′12″	37^0^26′23″	2285	Highland	7	8	7	8	13	2
	Loza Maryam	12^0^33′17″	37^0^26′16″	2155	Highland	7	8	5	10	14	1
	Maryam Debir	12^0^30′50″	37^0^25′0″	1979	Highland	5	10	5	10	14	1

Abbreviations: E, elder informants aged 54–87 years; F, female; Isl, Islamic followers; M, male; Ort, Ethiopian Orthodox Tewahido followers; Y, younger informants aged 20–53 years.

### 2.3. Data Collection Methods

Important ethnobotanical data were collected from October 2025 to February 2026 using semi‐structure interviews, guided field walks, focus group discussions, and market surveys following former authors [30, 31]. Individual interviews were conducted with all general and key informants individually using checklist of questions. It was applicable to collect demographic data (age, sex, education, occupation, marital status, and religion). It was also used to collect the main ethnobotanical data including WEPs local name, edible part, condition of consumption, mode of preparation, and also their sources of occurrence by referring Cotton [31].

Discussions were held with key informants in focus groups in order to get more information on WEPs. They were assembled at convenient places within their communities in order to verify, explain, and add to the information collected through personal interviews. The discussion was open‐ended so that the informants could share their information freely while the researchers facilitated the process as explained by Martin [30]. All valuable data were systematically documented during the interaction process, including comments by participants and observations by the researcher. Furthermore, in order to observe and identify WEPs in their natural habitats, guided field trips were done with certain informants. It was carried out in order to check the existence and distribution of WEPs, their growth forms and habitat, as well as the seasonality of the edible plant parts. Specimens and photographs were also obtained during the field visits to help in taxonomic identification. Additionally, market surveys were carried out in select markets to capture the commercial aspects of the WEPs, such as the units of measurements used, price levels, and marketing channels.

### 2.4. Plant Identification and Specimen Deposition

During the field expeditions, representative specimens of the WEPs were collected and delivered to the University of Gondar for taxonomic identification. The Department of Biology’s taxonomists assisted the identification of the plant species, and the Flora of Ethiopia and Eritrea served as the primary source of information. After the plants were identified, vouchers were prepared and numbered to be placed in the University Herbarium. The nomenclature of the plant taxa mentioned in the records was checked against the International Plant Names Index (IPNI) and the Plants of the World Online (POWO) database. Angiosperm Phylogeny Group classification system (APG IV) was followed for family identification task of the recorded plant species.

### 2.5. Ethnobotanical Data Analysis

Descriptive statistical methods were used to analyze the collected data, which were compiled and analyzed in Microsoft Excel 2007. Two‐tailed independent sample *t*‐test analysis (at 95% confidence interval) was performed to compare indigenous knowledge difference between general and key informants, urban and rural inhabitants and also between sex groups using SPSS software version 26.0. Pearson correlation coefficient was also used to analyze edible plants knowledge related to age. In addition, preference ranking and triadic comparison analysis techniques [30, 31] were employed to test consistency of responses and informant agreements as recommended.

Preference ranking was performed on seven most frequently mentioned WEPs based on taste using nine purposively selected key informants who have more knowledge on WEPs from the total 60 key informants. This number was chosen following the recommendation of Martin [30] and Cotton [31] in which 5–15 key informants are sufficient for preference ranking and other participatory ethnobotanical exercises.

In the preference ranking process, the informants assigned the numerical values between 1 and 7 to the listed species of plants, whereby the maximum value indicated the highest taste preference. These numerical values assigned by the informants were later summed up for each particular plant species, and the total number was used to determine the relative ranking of the species. This helped to identify the species that received the highest preference.

Regularity and connection of community’s preferences on WEPs based on safeness/comfort after mealing were analyzed using triadic comparison analysis method. To perform a feasible ranking, seven key informants were selected purposively following the minimum sample size requirement for triadic comparison (5–10 informants) [30, 31] and allowed them to select five edible plant species. The selected species were assigned by numbers, one to five, and then paired in to all possible triadic pairs using the pre‐assigned numbers. The possible numbers of triadic pairs were 10 which were calculated using the formula;

Number of triadic pairs=n!3!n−3!

where *n* is number of items (species) to be compared.

Jaccard’s coefficient of similarity (JCS) of WEPs species composition between the present study area and other 17 previous studies having similar agro‐ecologies was compared following Awas and Demissew [9]. JCS was calculated for paired habitat types (A and B) as follows:

JCS=cc+b+a

where *a* is the number of species found only in Habitat A, *b* is the number of species only in Habitat B, and *c* is the number of common species in Habitats A and B.

## 3. Results

### 3.1. Effect of Sociodemography on WEPs Knowledge

The analyzed result showed a statistically significant relationship between the sociodemographic features of the informants and the knowledge of WEPs (*p* < 0.005). There were also extremely significant differences regarding WEP knowledge depending on the place of residence of informants; both between urban and rural informants and between male and female informants (*p* < 0.001). Rural and male informant groups had higher indigenous knowledge with mean plant report of 8.89 and 8.62 WEPs, respectively (Table 2). The free plant list length of the study area also indicated a positive correlation between edible plants knowledge and age of respondents (r = 0.58, *p* < 0.05) with free plant list length ranged from 0 to 21 WEPs (Figure 2).

**Table 2:** Independent sample *t*‐test of significance on WEPs by informant groups.

Parameters	Informant group	*N*	No. of edible plants reported	Mean	Std. dev.	*t*value^**^	*p*value
Informant type	Key informants	60	522	8.70	4.49	−2.89	0.004
	General informants	120	813	6.78	4.08		
Study community	Rural community	90	800	8.89	4.56	4.87	0.000
	Urban community	90	535	5.94	3.48		
Sex	Male	89	767	8.62	4.54	3.84	0.000
	Female	91	568	6.24	3.73		

*Note:* Significant difference (*p* < 0.05), *N* = number of informants and std. dev. = Standard deviation, confidence interval = 95%.

^**^ *t* (0.05) two‐tailed.

**Figure 2:**
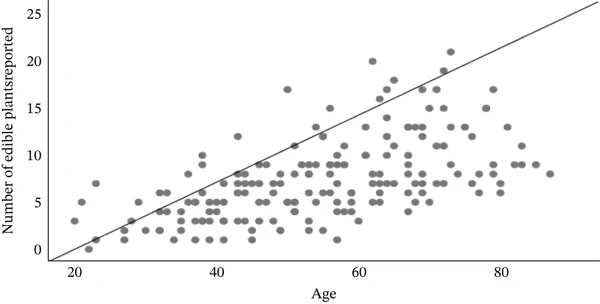
Scattered plot showing Pearson’s correlation between age and number of edible plants listed.

### 3.2. Taxonomic Diversity of WEPs

This study documented a total of 39 WEPs distributed in 32 genera and 23 families (Table 3). Both shrubs and trees were the most diversified plant habits each represented by 30.77% of the recorded WEPs whereas; climbers were the least (Figure 3). Fabaceae was the surpassed family represented by seven species (17.95%) afterward Moraceae with four species (10.26%), while 16 families were represented only by a single species each (Table 4). Most of the recorded species were dispersed in the wild natural habitat (64.1%) followed by both wild and homesteads (23.08%) whereas; least number of species (12.82%) was found only in homesteads.

**Table 3:** List of WEPs recorded in Gondar Administrative City.

Scientific name	Family	Habit	Edible part	Consumers	Harvesting time	No. of report	Consumption mode	Habitat	Added values	Voucher number
*Acanthus polystachyus* Delile	Acanthaceae	S	Fn	Ch	Nov–Dec	19	FR	W	Fr, Fw, Md	TA020
*Acanthus sennii* Chiov.	Acanthaceae	S	Fn	Ch	Nov–Dec	22	FR	W	Fr, Fw, Md	TA025
*Albizia schimperiana* Oliv.	Fabaceae	T	G	Ch	Year round	3	FR	W	Fw, Sh	TA003
*Amaranthus caudatus* L.	Amaranthaceae	H	L	All	Jun–Jul	7	FC	B	Fr	TA008
*Capparis tomentosa* Lam.	Capparaceae	Cl	F	Ch	Apr–May	12	FR	W	Md, Fw, Fc	TA014
*Carissa spinarum* L.	Apocynaceae	S	F	All	Apr–Jun	129	FR	W	Fc, Fr, Fw, Md	TA037
*Commelina benghalensis* L	Commenliaceae	H	R	Ch	May–Aug	17	FR	W	Fr	TA017
*Cordia africana* Lam.	Boraginaceae	T	F	Ch	Feb–Apr	138	FR, DR	Hs	At, Fr, Fw, Hu, Co, Md	TA038
*Cyperus rotundus L*	Cyperaceae	H	R	Ch	May–Jun	7	FR	W	Fr, Hb	TA009
*Datura stramonium* L.	Solanaceae	H	Fn	Ch	Aug–Sep	4	FR	B	Md	TA004
*Daucus carota* L.D. spp *carota*	Apiaceae	H	R	Ch	Mar–May	11	FR	W	Fr	TA013
*Dombeya torrida* (J.F. Gmel.) P. Bamps	Malvaceae	T	Sb	Ch	Year round	21	FR	W	Fr, Fw, Ro	TA023
*Dovyalis abyssinica* (A. Rich.) Warb.	Salicaceae	S	F	All	Jun–Aug	41	FR	Hs	Fc, Fw	TA032
*Dovyalis afra* (Hook.f. & Harv.) Warb.	Salicaceae	S	F	All	Jun–Aug	19	FR	B	Fc, Fw	TA021
*Ficus scycomorus* L.	Moraceae	T	F	Ch	Apr–May	17	FR, DR	W	Hu, Sh	TA018
*Ficus sur* Forssk	Moraceae	T	F	All	Feb–Apr	105	FR, DR	W	Md, Sh	TA035
*Ficus thonningii* Blume	Moraceae	T	F	Ch	Apr–May	5	FR	Hs	Fr, Fw, Sh, Md	TA007
*Ficus vasta* Forssk.	Moraceae	T	F	Ch	Jan–Mar	16	FR	W	Md, Sh	TA016
*Grewia ferruginea* Hochst. ex A. Rich.	Malvaceae	S	F	All	Dec–Feb	92	FR, DR	B	Fr, Fw, Md	TA034
*Mimusops kummel* A. DC.	Sapotaceae	T	F	All	Dec–Jan	26	FR	W	Sh	TA026
*Momordica foetida* Schumach.	Cucurbitaceae	Cl	F	All	Oct–Nov	37	FR	B	Md	TA030
*Opuntia ficus-indica* (L.)P. Mill.	Cactaceae	S	F	All	May–Aug	53	FR	Hs	Md, Fc	TA033
*Oxalis latifolia* Kunth,	Oxalicaceae	H	L	Ch	May–Jun	26	FR	W	—	TA027
*Physalis peruviana* L.	Solanaceae	H	F	Ch	Oct–Dec	27	FR	W	—	TA028
*Pterolobium stellatum* (Forssk.) Brenan	Fabaceae	Cl	F	Ch	Feb–Mar	4	FR	B	Md, Fc	TA005
*Rosa abyssinica* Lindle,	Rosaceae	S	F	All	Nov–Jan	114	FR	W	Fc, Fr, Fw, Md	TA036
*Rubus erlangeri* Engl.	Rosaceae	Cl	F	All	Feb–Apr	19	FR	W	Fr, Wc	TA022
*Rumex nervosus* Vahl	Polygonaceae	S	YS	Ch	Jun–Sep	8	FR	W	Fr, Fw, Md	TA011
*Searsia glutinosa* (Hochst. ex A.Rich.) Moffett ^∗^^**NE**^	Anacardiaceae	S	F	Ch	Aug–Sep	18	FR	W	Fr, Fw, Md	TA019
*Solanum villosum* L.	Solanaceae	H	F	All	Oct–Nov	38	FR	B	Md	TA031
*Sporobolus africanus* (Poir) Robyns and Tournay	Poaceae	H	S	All	Oct–Nov	2	DB	W	Fr, Br	TA001
*Syzygium guineense* (Willd) DC.	Myrtaceae	T	F	All	May–Jun	157	FR	W	Fw, Sh, Wc	TA039
*Urtica simensis* Steudel	Urticaceae	H	L	All	Jun–Jul	2	FC	B	Ro	TA002
*Vachellia abyssinica* (Hochst. ex Benth.) Kyal. & Boatwr.	Fabaceae	T	Sb, G	Ch	Year round	33	FR	B	Ch, Fr, Fw, Sh, Md	TA029
*Vachellia nilotica* L Hurter & Mabb.	Fabaceae	T	G	Ch	Year round	4	FR	W	At, Ch, Fr, Md	TA006
*Vachellia seyal* (Delile) P.J.H.Hurter.	Fabaceae	T	G	Ch	Year round	7	FR	W	Ch, Fw, Fc, Fr	TA010
*Vigna membranacea* A.Rich.	Fabaceae	Cl	R	Ch	May–Jun	9	FR	W	—	TA012
*Ximenia Americana* L.	Olacaceae	S	F	All	Mar–Apr	12	FR	W	Md	TA015
*Ziziphus spina-christi* (L.) Desf.	Rhamnaceae	S	F	All	Feb–Apr	21	DR	W	Md, Fr, Fw	TA024

*Note:* Habit; T (tree), S (shrub), H (herb), Cl (climber); Edible plant part; F (fruit), G (gum), L (leaf), Fn (Flower nectar), R (root), S (seed), Sb (stem bark), Ys (young stem); Mode of consumption; FR (fresh raw), DR (dried raw), FC (fresh cooked), DB (dried baked); Consumers; Ch (children), All (all individuals); Added values; At (agricultural tools), Br (Broom), Co (construction), Ch (Charcoal), Fc (fencing), Fr (fodder), Fw (fire wood), Hb (house beauty), Hu (household utensil), Sh (shading), Md (traditional medicine), Ro (rope), Wc (water conservation); Habitat; W (wild), Hs (homestead), B (both wild and homestead).

**Figure 3:**
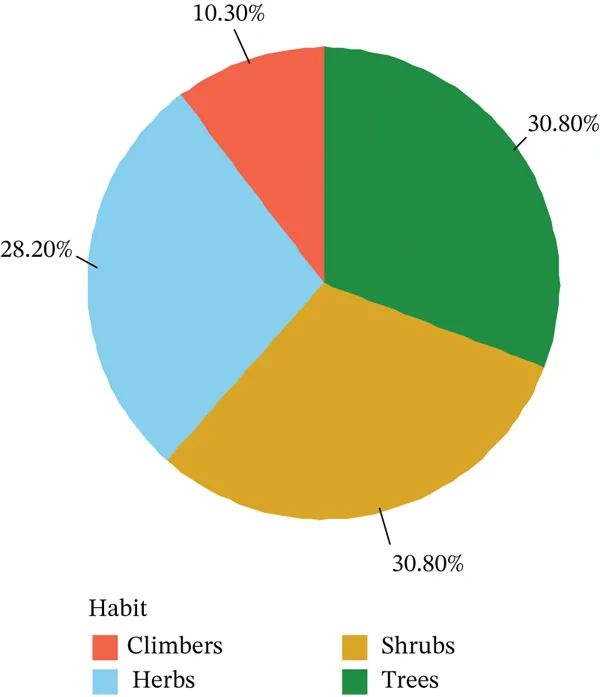
Growth forms of WEPs.

**Table 4:** Families of WEPs in the study area.

Family	Number of genera	Number of species	Percentage
Fabaceae	5	7	17.95
Moraceae	1	4	10.26
Solanaceae	3	3	7.69
Malvaceae	3	3	7.69
Rosaceae	2	2	5.13
Acantaceae	1	2	5.13
Salicaceae	1	2	5.13
16 families	1	1	2.56

The use value reports revealed that six species were informed by more than half of the total informants. Of which, *Syzygium guineense* was the most common WEP with 87.22% consensus followed by *Cordia africana* with 76.67% common agreement. Whereas, *Urtica simensis* and *Sporobolus africanus* were reported only by two informants each for their edible value (Table 3). Preference ranking analysis also showed that, *Cordia africana* was the tastiest WEPs followed by *Syzygium guineense* while *Grewia ferruginea* was the least (Table 5). Triadic comparison analysis indicated *Syzygium guineense* as the safest WEP after mealing followed by *Ximenia americana* whereas; *Ficus sur* ranked least (Table 6).

**Table 5:** Taste based preference ranking of seven more frequently listed WEPs.

Plant species	Informants									Sum	Rank
	I1	I2	I3	I4	I5	I6	I7	I8	I9		
*Cordia africana*	6	5	6	7	6	7	7	6	5	55	1st
*Syzygium guineense*	7	6	7	5	5	6	6	7	4	53	2nd
*Carissa spinarum*	5	7	4	4	7	5	5	4	7	48	3rd
*Ficus sur*	4	4	3	6	3	4	4	5	3	36	4th
*Opuntia ficus-indica*	3	3	1	3	4	2	3	3	2	24	5th
*Rosa abyssinica*	2	1	2	2	1	3	2	1	6	20	6th
*Grewia ferruginea*	1	2	5	1	2	1	1	2	1	16	7th

**Table 6:** Triadic comparison of five selected WEPs based on safeness after mealing.

Plant species	Informants							Sum	Rank
	I1	I2	I3	I4	I5	I6	I7		
*Syzygium guineense*	3	3	1	2	5	5	3	22	1st
*Ximenia americana*	3	1	0	6	4	3	1	18	2nd
*Opuntia ficus-indica*	3	5	0	1	0	1	1	11	3rd
*Carissa spinarum*	1	0	6	0	1	1	1	10	4th
*Ficus sur*	0	1	3	1	0	0	4	9	5th

### 3.3. Jaccard’s Similarity Index (JSI)

A comparison with 17 previous publications yielded Jaccard similarity percentages of 2.67%–50% in the traditional knowledge of WEPs. Communities of the current study area had the highest similarity (50% similarities) in terms of the indigenous knowledge with the communities of Sedie Muja District followed by communities of Simada District with 33.33% resemblance (Table 7). On the other hand, the lowest likeness level of indigenous knowledge on WEPs was observed in relation to communities living in the Omo River Valley region, with a JSI of 2.65%.

**Table 7:** Jaccard’s similarity index between the current study area and previous reports.

Study area	WEPRPA	a	b	c	JSI (%)	Source
Simada	45	18	24	21	33.33	[31]
Dheerar Town	40	26	25	18	26.09	[8]
Awi	39	29	29	10	14.71	[11]
Ensaro	43	21	25	18	28.13	[32]
Liben and Wareda	54	25	40	14	17.72	[33]
Adola	46	23	30	16	23.19	[34]
Tach Gayint	36	21	18	18	31.58	[35]
Sidama	50	29	40	10	12.66	[36]
Chilga	33	25	19	14	24.14	[37]
Omo River Valley	38	37	36	2	2.67	[38]
Soro	64	21	46	18	20.45	[438]
Midakegn	50	22	33	17	23.61	[39]
Dibatie	54	28	43	11	13.41	[40]
Sedie Muja	33	15	9	24	50.00	[41]
Burji	46	30	37	9	11.84	[42]
Quara	51	27	39	14	17.50	[43]
Metema	44	27	32	12	16.90	[19]

Abbreviations: a, wild edible plant species unique to current study area; b, WEPs unique in the previous study area; c, WEPs common in both study areas; JSI (%), Jaccard’s similarity index in percent; WEPRPA, total wild edible plants recorded in the previous study area.

### 3.4. Plant Parts Used and Mode of Consumption

Fruits were the prominent edible plant parts which accounted 56.41% and then roots with 10.26% of the total. In contrast, young stem, stem bark, and seeds were the least edible plant parts each accounted 2.56% (Figure 4). Most (79.49%) of the edible plant parts were consumed in fresh and raw forms without any processing, while dried baked and dried raw forms were the least consumption modes each with 2.56% (Figure 5).

**Figure 4:**
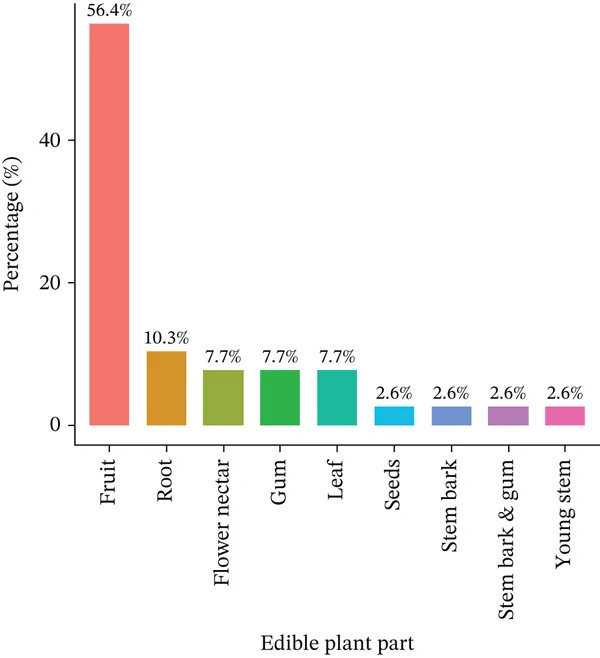
Edible plant parts and their mean percentage.

**Figure 5:**
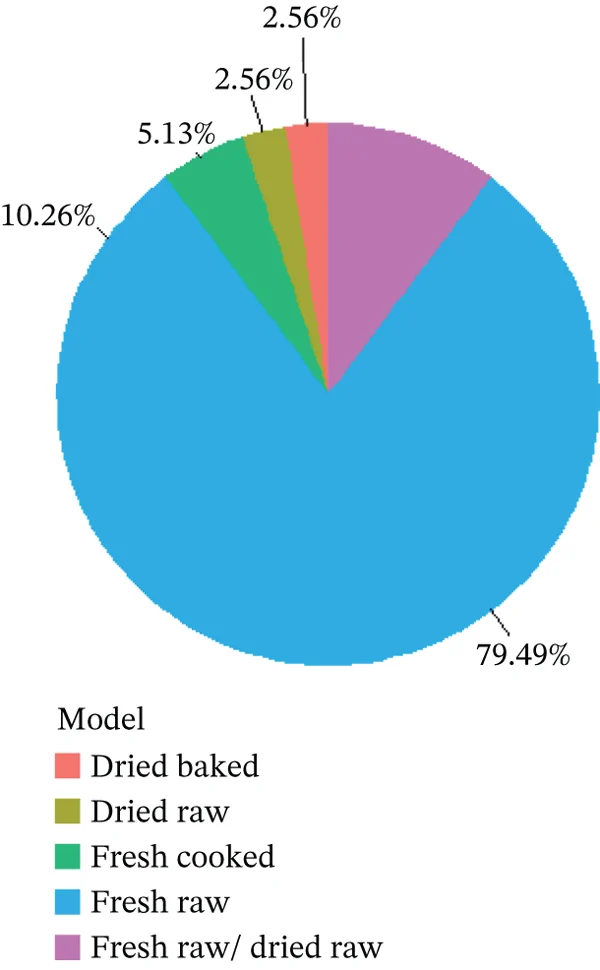
Mode of consumption of WEPs.

### 3.5. WEPs in Household Food Security

Majority of identified WEPs (92.31%) played a supplementary role in food security, as they were primarily consumed as snacks or refreshment foods when ample food was available rather than being considered as emergency food sources. Of those, about 58.97% were consumed by youngsters aged below 20 mainly by livestock herders to fill their daily food gap in the field. The remaining 35.89% were used by all age groups when accessible (Table 3). Three species (*Urtica simensi*, *Amaranthus caudatus*, and *Sporobolus africanus*) were the only edible plants reported as emergency foods that were consumed during famine periods.

### 3.6. Seasonal Availability and Use Diversity of WEPs

From the recorded WEPs, 12.82% were accessible throughout the year, whereas, 87.18% were harvested during specific month ranges. Highest number of WEPs (41.03%) was harvested in June and then in May (38.46%). Conversely, lower numbers of WEPs (20.51%) were accessible in September (Table 8). Apart from their edible value, most of the identified WEPs (92.31%) had diversified purposes. Of which, 53.8% were used as traditional medicine and 48.7% for livestock fodder (Figure 6). From those, *Cordia africana* was reported as the most multipurpose species with seven use values (Table 3).

**Table 8:** Harvesting time of WEPs in the study area.

Harvesting time	Number of accessible WEPs	Percentage
January	9	23.07
February	11	28.21
March	13	33.33
April	14	35.89
May	15	38.46
Jun	16	41.03
July	12	30.77
August	12	30.77
September	8	20.51
October	9	23.07
November	11	28.21
December	10	25.64

*Note:* Each edible plant part might be accessible year round or for months (see Table 3).

**Figure 6:**
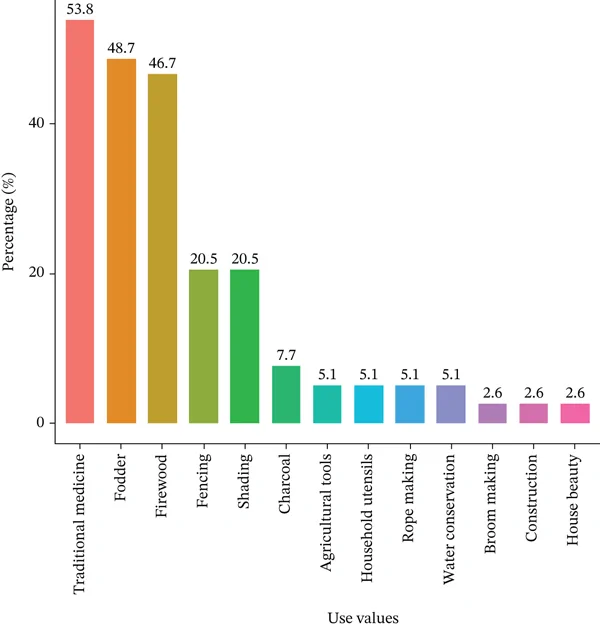
Additional uses of WEPs.

### 3.7. Marketability of WEPs

In addition to their household consumption, fruits of four species (*Mimusops kummel*, *Syzygium guineense, Ziziphus spina-christi*, and *Opuntia ficus-indica*) were sold as food in local markets of Gondar City (Figure 7). Measuring units of *Mimusops kummel*, *Syzygium guineense*, and *Ziziphus spina-christi* were cups with mean unit price of 25, 12.5, and 10 Ethiopian birr (ETB), respectively. While, *Opuntia ficus-indica* was sold in fruit count or kilogram (kg) with mean average price of 10 ETB per fruit or 50 ETB per kg. The market chain of these WEPs is relatively short and involves collectors, vendors, and consumers. These edible plants were gathered mostly by male children and youth from their natural habitat. Then, retailers (mainly female from poor families) bought the fruits from the collectors and then sold in the local markets.

**Figure 7:**
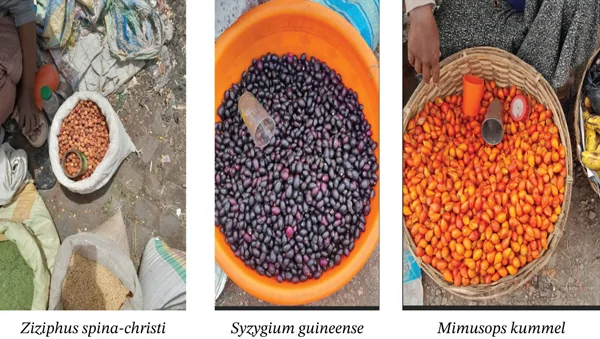
Fruits of WEPs sold in Gondar City Streets. *Ziziphus spina-christi, Syzygium guineense, Mimusops kummel.*

## 4. Discussion

### 4.1. Effect of Sociodemography on WEPs Knowledge

Independent sample *t*‐test analysis confirmed the presence of significant sociodemographic effect on WEPs knowledge in the area (*p* < 0.05). This corroborated with other findings in the country [32, 39, 40], which is an indicator for uneven knowledge distribution among different informant groups across Ethiopia. Differences in the degree of interaction of informant’s with plants might be the cause of unequal knowledge sharing. Urban communities were found less knowledgeable than rural communities, which might be related to modernization since it was reported as one of the big threats for indigenous knowledge loss [14, 44]. Likewise, the absence of direct and lifelong interaction between urban communities and the natural vegetation [40, 45] might make them less knowledgeable than rural communities.

Significantly higher knowledge of males over females was observed in this study, which contradicts the report of previous studies [45–47]. It was reported that, in the rural communities of most developing countries women get involved in multiple household activities including collection of firewood, fodder, food plants and also fetching of water over a long distance [24, 44, 46, 48]. However, in the current study area, the involvement of females in the above mentioned activities are limited only in the rural kebeles within a short distance from their home. Data obtained from individual interviews and focus group discussions confirmed that males frequently spent most of their time in contact with natural vegetation through cattle herding, swimming or other refreshment activities. Therefore this study highlights the importance of considering local socio‐cultural dynamics in ethnobotanical studies for the distribution of indigenous knowledge.

A positively correlated relationship between age and knowledge of WEPs was also observed in other previous findings [43, 44]. This might be linked with long lasting, regular and direct contact of elders with edible plants which provided them to accumulate more knowledge. As some elder informants stated during focus group discussions, they grew up while eating many WEPs while their children only know the names but have never tasted most of them either due to modernization or loss of the plants in the local area. Most elder informants also informed that they consumed most of the WEPs during the childhood (Table 3) but not consumed them while aging either due to cultural influence or loss of the species in the area. This indicated that the loss of WEPs and the indigenous knowledge might be aggravated in the near future unless immediate plant conservation and proper knowledge documentation activities are taken as reported before [40, 49].

### 4.2. Taxonomic Diversity of WEPs

Considerable numbers of WEPs were recorded in the study area, revealed that towns and their surroundings are homes for WEPs, which are comparable with other rural environments. The numbers of edible plants recorded are higher than previous reports conducted in different rural areas of the Ethiopia [36, 38, 42, 50, 51] but lower than those reported in other parts of the country [19, 32, 40, 48]. Many species documented in this study were also documented elsewhere in Ethiopia; to site some, 14 species in the Awi Agäw community [11], 20 species in Simada District [32], 16 species in Ensaro District [33], 14 species in Tach Gayint District [36], 13 species in Chilga District [38], and nine species in Awulaelo District [52]. The presence of these plants in various parts of the country demonstrates their ecological adaptation across a wide geographical range and their suitability for consumption by diverse ethnic groups, which is an indicator for the role of WEPs in food security across the country.

Predominance of trees and shrubs among the documented WEPs is an indicator for the contribution of woody vegetation as principal edible plant sources, which are in line with many previous studies in Ethiopia [13, 19, 40, 53, 54]. A review conducted by Lulekal et al. [2] also revealed shrubs and trees as the dominant WEP habits, which accounted 31% and 30% of the total edible plants of the country, respectively. Data collected during, focus group discussions and field observations through guided field walks also confirmed the presence of many edible trees and shrubs within farm lands as agroforestry systems and also in boundaries. These were primarily conserved by farmers for their multiple roles including household utensils, agricultural tools, firewood, and living fencings. They were also distributed in church compounds as sacred vegetation, city road sides, and homesteads. These traditional conservation methods along with the species longer period of survival might made them to be the dominant edible plant habits in the area as reported by former authors [5, 53].

At family level, the predominance of Fabaceae might be allied with its successfulness in species diversity and ecological adaptability across a wide range of tropical and subtropical regions [53, 55]. So, the ability of the family to survive in heterogeneous habitats could be the main reason why species under Fabaceae are commonly used as WEPs in the study site. A national‐level survey by Tadesse et al. [16] also informed Fabaceae as the most abundant plant family among the WEPs in Ethiopia. Moraceae were the second leading family in this study while, it was reported as the fifth family across Ethiopia [16]. It was also reported as the second family with many WEP species in different studies [39, 42, 44], showing its ecological importance range in different local communities. In contrast, the occurrence of many families represented by only a single species suggests a broad taxonomic diversity of WEPs and independency of local communities to utilize food resources from a few dominant families. The species are widely distributed in wild habitat which is in line with previous reports [40, 53]. This highlights the role of wild ecosystems in harboring WEPs and supporting the livelihood of local communities. The availability of heterogeneous habitats in the wild environment which facilitate the co‐existence of many plant species might be the cause for their wilder occurrence. Conversely, the limited number of species confined exclusively to homesteads demonstrates that domestication steps of WEPs remains relatively limited in the study area. As data gathered during interview, less number of WEPs recorded in homesteads were not grown primary for their edible value but for other uses or grown as weeds. This is an indicator for the need of creating awareness among local communities about the conservation and cultivation of WEP species in homesteads to promote their use as alternative food sources for sustainable food security.

The difference in frequency of citation of WEPs might be connected with uneven geographical distribution of the plants and/or knowledge sharing variation between the communities. Based on the report of Xie et al. [56], the citation frequency of plant species for a specific use value reflects their relative importance within the local community and species with the highest report are considered the most valuable for that specific use. Accordingly, *Syzygium guineense* is expected to be consumed by many informants followed by *Cordia africana*. This is supported by triadic comparison analysis result in which *Syzygium guineense* is the most preferring and relaxing WEP. Sintayo Demise and Zebene Asfaw [35] also reported *Syzygium guineense* as the first preferred edible plant by Adola District communities. But it was ranked last from six edible plants by communities of Ensaro District [44]. This might be associated with variations in nutritional proportion of its fruits due to edaphic or other factors in the areas. It might also be related with variation in long lasting community perceptions.

### 4.3. JSI

The observed difference in JSI indicated the presence of meaningful difference in the indigenous knowledge distribution and utilization of WEPs between Ethiopian communities. Relatively the highest knowledge similarity of current study community with communities of Sedie Muja [42] and Simada [32] Districts might be related to closer area of proximity in between with comparable floristic composition, which allows sharing of agroecological conditions, cultural traditions, and the ethnobotanical knowledge. Their similar ecological region (The Gondar Region) and their relative cultural proximity might also facilitate social interactions and exchange of indigenous knowledge which contribute to greater overlap in the diversity and use of WEPs. In contrast, results obtained from the study in the Omo River Valley [57] revealed that variability in environment and culture within the region is capable of shaping the WEPs composition as well as the traditional knowledge about their use. The Omo River Valley is different in climate, vegetation, and cultural/ethnic background from northwest Ethiopia. These variations might be the reason behind differences in the availability and use of the WEPs and aboriginal knowledge about them. This highlights Ethiopian populations’ immense cultural diversity and widespread consumption of WEPs. Studies have also shown that the diversity and type of indigenous knowledge systems are significantly shaped by cultural traits and geographic environment [6, 8, 30, 40]. This highlights the need of documenting indigenous knowledge across all the diverse ecological and cultural settings of the country before their total lose through environmental shifting, urbanization and sociocultural transformation.

### 4.4. Plant Parts Used and Mode of Consumption

The prevalence of fruits as edible plant species in the current study is in line with findings reported in different studies carried out in Ethiopia [39, 40, 53, 54] and in China [38]. Information from focus group discussion data confirmed that the preference for fruits is mainly because of their palatability, easy accessibility, and immediate consumption after picking. So, the lack of need for any preparation might make them more valuable, especially for children and those individuals who are involved in their daily work activities like farming and livestock rearing and thus spend most of their time outside their homes [19, 25]. Fruit harvesting was also found to be a sustainable activity since the collection of fruits did not entail the destruction of roots, stems, or even whole plants. Therefore, the parent plants remained intact and continued to provide edible resources in their natural settings.

The pattern found in the current research study is different from those found in some parts of China [58] and Iran [59], where leaves were the main edible parts of plants. Regional differences can be caused by differences in food culture, climate, and information passed on from generation to generation, as was indicated by Baressa Anbessa [13]. In the current study area, most of the WEPs were consumed in their fresh state without being cooked or processed, which is consistent with other studies [11, 32, 35]. The consumption of WEPs in their fresh state can be explained by the desire to preserve the natural taste and properties of the plants that were thought to change after cooking or processing. Lack of immediate access to cook after collection might also be a reason which enforced them to develop a habit of consuming unprocessed fresh forms since most of the edibles are consumed by cow herders in the wild.

### 4.5. WEPs in Household Food Security

Most of the recorded WEPs were consumed in the presence of ample food which is an indication for their supplementary role which is in line with former studies in Ethiopia [13, 39, 40, 54] and other country [60]. Informants were highly enforced to consume the edibles to fill their daily food gaps or as a refreshment foods. As reported, during the ripening periods of *Carissa spinarum*, *Ficus sur*, and *Syzygium guineense* livestock herders of rural communities did not get hungry trough eating the ripped fruits and when they returning to home their need to ate food is not as much as the usual. In contrary, other studies in different areas of Ethiopia [53, 57] have reported that most WEPs are consumed as famine foods. This implied that, consumption of WEPs might be more dependent on access of normal meals in the area, reflecting availability of better food security in the current study area compared with other drought prone areas of Ethiopia.

### 4.6. Seasonal Availability and Use Diversity of WEPs

Seasonal availability of WEPs was directly related to growth form, edible plant part and the nature of the species. Perennial plants with edible gum and internal stem barks were accessible throughout the year however while, edible herbs were available during or sometimes after the rainy season. Higher accessibility of edible fruits from trees and shrubs during the months of May and June were consistent with previous report [53]. As David and Kobe [61] reported, trees fruiting can peak in March and April with most fruits ripening from May to July. The highest availability of these WEPs in the crops’ pre‐harvesting time of many parts of Ethiopia is correspondent with the “hunger season” highlights the need of incorporating WEPs into the normal food staff.

The multiple uses of most identified WEPs other than food indicated the diversified indigenous knowledge of the community on a given plant species. This corroborate with the report of Daniel Tadesse et al. [48]. However, the higher use diversity of the species might result in over exploitation that would lead to loss of the species in the near future as Gemedo Dalle et al. [62] stated.

### 4.7. Marketability of WEPs

Some of the recorded WEPs are not limited only on direct food source but had cash income generating role. It is an alternative income sources to meet the basic needs of the poor families, which is in line with former report [40]. Compared with previously reported studies carried out on other regions of Ethiopia [11, 38, 48, 53], the current study has observed the presence of fewer species of WEPs that have some market values. It should be noted that all the four species recorded in the current study region for their commercial importance have already been recognized elsewhere as providing livelihood incomes. To indicate some, *Opuntia ficus-indica* and *Ziziphus spina-christi* in Chilga District [38] and Awulaelo District [52], *Syzygium guineense* and *Ziziphus spina-christi* in Metema [19] and Quara [50] districts and *Syzygium guineense* and *Mimusops kummel* by Awi communities [11]. This revealed that WEPs could have significant cash income value to the country if conservation priorities are given. The relatively short market chain, involving collectors, retailers, and consumers, suggests that the trade in WEPs is largely informal and locally organized. Such simplified marketing systems enable producers and collectors to sell products quickly with minimal transaction costs. However, the localized market chain might limit the chance of knowledge sharing between communities, decrease the diversification of WEPs, and restrict higher financial exchanges.

## 5. Conclusions

This study assessed the diversity, indigenous knowledge, utilization, and marketability of WEPs in rural and urban communities of Gondar Administrative City. It documented 39 WEP species distributed across 25 families, demonstrated that urban and semi‐urban areas harbor considerable WEP diversity comparable with rural areas. This challenging the common assumption that useful plant knowledge is confined to rural communities and emphasized highly scattered urban vegetation along roadsides, green areas, and homesteads to have a considerable role in food security. The study also reflected a significant urban‐rural knowledge gap (*p* < 0.001) and positive correlation between age and knowledge (r = 0.58) highlights the urgent need for documentation and preservation efforts particularly in urban areas, where modernization and changed lifestyles reduce exposure to WEPs. Both trees and shrubs were the common WEP habits (30.77%) and most of the recorded species were dispersed in the wild natural habitat (64.1%). This indicated that conserving natural woody vegetation is essential for maintaining WEPs diversity and ensuring their continued contribution to household food security and rural livelihoods. Conservation priorities should focused on protecting remaining natural habitats, particularly church forests which serve as refugia for WEPs, cultivation of high‐value WEPs in homesteads and developing sustainable harvesting guidelines to prevent overexploitation of high value species like *Cordia africana* and *Syzygium guineense*. Conservation actions should also be integrated WEPs into broader urban biodiversity strategies and green corridors which could provide natural habitats within the city. In addition, partnerships among government, research institutions, private companies and local communities should be established by identifying potentially valuable WEPs for their sustainable commercialization through value addition, improved market access, and income generation.

## Author Contributions

**Tiruye Ayenew**: conceptualization, methodology, data curation, investigation, formal analysis, writing original draft, and writing – review and editing. **Abiyu Enyew**: conceptualization, validation, supervision. **Getinet Masresha**: supervision, funding acquisition, and writing – review and editing. **Worku Misganaw**: validation, formal analysis, and writing – review and editing. **Daniel Tadesse**: conceptualization and validation. **Eshete Kassaw**: writing – review and editing. **Birtukan Asrie**: data curation. **Yirgalem Melkamu**: conceptualization, methodology, validation, formal analysis, supervision, and writing – review and editing.

## Funding

This study was supported by University of Gondar, 10.13039/501100007861.

## Disclosure

The submitted manuscript was examined and approved by all authors.

## Ethics Statement

This research was conducted with ethical approval obtained from the College of Natural and Computational Sciences, University of Gondar (clearance number CNCS/02/03/11/09/2025). After obtaining the ethical approval letter, the nature and rationale of this research were explained to all participants and unrecorded verbal consent was obtained prior to data collection.

## Conflicts of Interest

The authors declare no conflicts of interest.

## Data Availability

All the necessary data collected for this study are analyzed, interpreted and included in this manuscript.
